# Supplementary material for: From sequence to enzyme mechanism using multi-label machine learning
Source: BMC Bioinformatics. 2014 May 19;15:150. doi: 10.1186/1471-2105-15-150 (PMC4229970; doi:10.1186/1471-2105-15-150)
Supplement: Additional file 2 — Java code of ml2db. Additional file ml2db_code.tar.gz contains the Java source code to run the multi-label machine learning experiments and save the results to database. The code’s Javadoc is included. [file 1471-2105-15-150-S2.zip › additional file 2/ml2db/ml2db/doc/constant-values.html]

Constant Field Values


JavaScript is disabled on your browser.


- Overview
- Package
- Class
- Use
- Tree
- Deprecated
- Index
- Help

- Prev
- Next

- Frames
- No Frames

- All Classes

# Constant Field Values

## Contents

- main.ml2db.\*
- test.ml2db.\*

## main.ml2db.\*

- main.ml2db.ProjectParameters

  | Modifier and Type | Constant Field | Value |
  | --- | --- | --- |
  |  |  |  |
  | --- | --- | --- |
  | `public static final java.lang.String` | `ARFF_FILES_DIR` | `"arff/"` |
  | `public static final java.lang.String` | `ARFF_FILES_PATH` | `"data/main/arff/"` |
  | `public static final java.lang.String` | `ARFF_PROPS_PATH` | `"data/main/properties_files/arff_props/"` |
  | `public static final java.lang.String` | `ARFF_TABLE` | `"arff"` |
  | `public static final java.lang.String` | `AUTHOR` | `"Luna De Ferrari - ldf(at)st-andrews.ac.uk"` |
  | `public static final java.lang.String` | `DATA_LOCAL_DB` | `"data/main/properties_files/machine_learning_props/mulan_data_local_server.props"` |
  | `public static final java.lang.String` | `DATA_REMOTE_DB` | `"data/main/properties_files/machine_learning_props/mulan_data_remote_server.props"` |
  | `public static final int` | `EXPECTED_NUMBER_OF_UNIQUE_ATTRIBUTES` | `20000` |
  | `public static final int` | `EXPECTED_NUMBER_OF_UNIQUE_INSTANCES` | `300000` |
  | `public static final java.lang.String` | `FIGURES_PATH` | `"data/main/figures/"` |
  | `public static final java.lang.String` | `MACHINE_LEARNING_LOCAL_DB_PROPS` | `"data/main/properties_files/machine_learning_props/mulan_results_local_server.props"` |
  | `public static final java.lang.String` | `MACHINE_LEARNING_PROPS` | `"data/main/properties_files/machine_learning_props/mulan_results_remote_server.props"` |
  | `public static final java.lang.String` | `MACHINE_LEARNING_REMOTE_DB_PROPS` | `"data/main/properties_files/machine_learning_props/mulan_results_remote_server.props"` |
  | `public static final java.lang.String` | `MACIE_HOM_DB_PROPS` | `"data/main/properties_files/machine_learning_props/mulan_data_local_macie_homologs.props"` |
  | `public static final java.lang.String` | `MODELS_PATH` | `"data/main/model/"` |
  | `public static final java.lang.String` | `PREDICTIONS_PATH` | `"data/main/predictions/"` |
  | `public static final java.lang.String` | `PROJECT_NAME` | `"ml2db"` |
  | `public static final java.lang.String` | `RESULTS_DIRECTORY` | `"data/main/results/"` |
  | `public static final java.lang.String` | `RESULTS_DIRECTORY_ACTIVE_LEARNING` | `"data/main/results/active_learning/"` |
  | `public static final java.lang.String` | `SOFTWARE_PROJECT_NAME` | `"ml2db"` |
  | `public static final java.lang.String` | `STATISTICS_PATH` | `"data/main/statistics/"` |
  | `public static final java.lang.String` | `UNIPROT_DB_PROPS` | `"data/main/properties_files/machine_learning_props/mulan_data_local_uniprot.props"` |
  | `public static final java.lang.String` | `VERSION` | `"2014_02"` |
  | `public static final java.lang.String` | `WEB_PAGE` | `"http://sourceforge.net/projects/ml2db/"` |

- main.ml2db.al.ActiveLearning

  | Modifier and Type | Constant Field | Value |
  | --- | --- | --- |
  |  |  |  |
  | --- | --- | --- |
  | `public static final java.lang.String` | `EUCLIDEAN_DISTANCE_NAME` | `"Euclidean distance"` |

- main.ml2db.al.database.AlDbCreator

  | Modifier and Type | Constant Field | Value |
  | --- | --- | --- |
  |  |  |  |
  | --- | --- | --- |
  | `public static final java.lang.String` | `EXPERIMENT_ID` | `"experiment_id"` |
  | `public static final java.lang.String` | `INSTANCE` | `"instance"` |
  | `public static final java.lang.String` | `STEP_ID` | `"step_id"` |
- main.ml2db.al.database.AlEvaluationTable

  | Modifier and Type | Constant Field | Value |
  | --- | --- | --- |
  |  |  |  |
  | --- | --- | --- |
  | `public static final java.lang.String` | `EVALUATION_TABLE_NAME` | `"evaluation"` |
- main.ml2db.al.database.AlExperimentsTable

  | Modifier and Type | Constant Field | Value |
  | --- | --- | --- |
  |  |  |  |
  | --- | --- | --- |
  | `public static final java.lang.String` | `ALGORITHM` | `"algorithm"` |
  | `public static final java.lang.String` | `DISTANCE_FUNCTION` | `"distance_function"` |
  | `public static final java.lang.String` | `END_TIMESTAMP` | `"end_time"` |
  | `public static final java.lang.String` | `EXPERIMENTS_TABLE_NAME` | `"experiments"` |
  | `public static final java.lang.String` | `LABELLED_ARFF_ID` | `"labelled_arff_id"` |
  | `public static final java.lang.String` | `START_TIMESTAMP` | `"start_time"` |
  | `public static final java.lang.String` | `UNLABELLED_ARFF_ID` | `"unlabelled_arff_id"` |
  | `public static final java.lang.String` | `UTILITY_FUNCTION` | `"utility_function"` |
- main.ml2db.al.database.QueryTable

  | Modifier and Type | Constant Field | Value |
  | --- | --- | --- |
  |  |  |  |
  | --- | --- | --- |
  | `public static final java.lang.String` | `INSTANCE_STRING` | `"instance_string"` |
  | `public static final java.lang.String` | `QUERY_TABLE_NAME` | `"query_instances"` |
- main.ml2db.al.database.UtilityTable

  | Modifier and Type | Constant Field | Value |
  | --- | --- | --- |
  |  |  |  |
  | --- | --- | --- |
  | `public static final java.lang.String` | `UTILITY_FUNCTION` | `"utility_function"` |
  | `public static final java.lang.String` | `UTILITY_TABLE_NAME` | `"utility"` |

- main.ml2db.al.utility.confidence.ConfidenceMetric

  | Modifier and Type | Constant Field | Value |
  | --- | --- | --- |
  |  |  |  |
  | --- | --- | --- |
  | `public static final boolean` | `m_debug` | `false` |
  | `public static final java.lang.String` | `NAME` | `"Utility based on uncertainty"` |

- main.ml2db.al.utility.globaldistance.DistancesStorage

  | Modifier and Type | Constant Field | Value |
  | --- | --- | --- |
  |  |  |  |
  | --- | --- | --- |
  | `public static final java.lang.String` | `DIVIDER` | `"\|"` |
- main.ml2db.al.utility.globaldistance.GlobalDistanceMetric

  | Modifier and Type | Constant Field | Value |
  | --- | --- | --- |
  |  |  |  |
  | --- | --- | --- |
  | `public static final java.lang.String` | `NAME` | `"Global distance metric + 1"` |
  | `public static final double` | `STANDARD_BALANCE` | `0.5` |
- main.ml2db.al.utility.globaldistance.GlobalPositionStore

  | Modifier and Type | Constant Field | Value |
  | --- | --- | --- |
  |  |  |  |
  | --- | --- | --- |
  | `public static final int` | `LABELLED_ROLE` | `0` |
  | `public static final int` | `UNLABELLED_ROLE` | `1` |

- main.ml2db.al.utility.hybrid.GlobalDistanceWithConfidenceMetric

  | Modifier and Type | Constant Field | Value |
  | --- | --- | --- |
  |  |  |  |
  | --- | --- | --- |
  | `public static final java.lang.String` | `NAME` | `"Global distance metric with confidence"` |

- main.ml2db.al.utility.neighbours.NeighbourhoodNetwork

  | Modifier and Type | Constant Field | Value |
  | --- | --- | --- |
  |  |  |  |
  | --- | --- | --- |
  | `public static final int` | `NUMBER_OF_NEIGHBOURS_1` | `1` |

- main.ml2db.al.utility.pickers.byfrequency.NearestNeighboursByFreqUtility

  | Modifier and Type | Constant Field | Value |
  | --- | --- | --- |
  |  |  |  |
  | --- | --- | --- |
  | `public static final java.lang.String` | `NEIGHBOURHOOD_BASE_NAME` | `"nn"` |

- main.ml2db.graphviz.GraphViz

  | Modifier and Type | Constant Field | Value |
  | --- | --- | --- |
  |  |  |  |
  | --- | --- | --- |
  | `public static final java.lang.String` | `DOT` | `"dot"` |
  | `public static final java.lang.String` | `DOT_COMMENT_PREFIX` | `"\n//"` |
  | `public static final java.lang.String` | `DOT_UNDIRECTED_EDGE` | `"--"` |
  | `public static final java.lang.String` | `FIG` | `"fig"` |
  | `public static final java.lang.String` | `GIF` | `"gif"` |
  | `public static final java.lang.String` | `JPG` | `"jpg"` |
  | `public static final java.lang.String` | `PDF` | `"pdf"` |
  | `public static final java.lang.String` | `PLAIN` | `"plain"` |
  | `public static final java.lang.String` | `PNG` | `"png"` |
  | `public static final java.lang.String` | `PS` | `"ps"` |
  | `public static final java.lang.String` | `SVG` | `"svg"` |
- main.ml2db.graphviz.GraphVizArff

  | Modifier and Type | Constant Field | Value |
  | --- | --- | --- |
  |  |  |  |
  | --- | --- | --- |
  | `public static final java.lang.String` | `BASE_ATTRIBUTE_SHAPE` | `""` |
  | `public static final java.lang.String` | `BASE_CLASS_SHAPE` | `" [shape=triangle]"` |
  | `public static final java.lang.String` | `BASE_INSTANCE_SHAPE` | `" [shape=box]"` |
- main.ml2db.graphviz.GraphVizArffColoured

  | Modifier and Type | Constant Field | Value |
  | --- | --- | --- |
  |  |  |  |
  | --- | --- | --- |
  | `public static final java.lang.String` | `LABELLED_ATTRIBUTE_SHAPE` | `" [style=filled,color=cyan]"` |
  | `public static final java.lang.String` | `LABELLED_CLASS_SHAPE` | `" [shape=triangle,style=filled,color=cyan]"` |
  | `public static final java.lang.String` | `LABELLED_INSTANCE_SHAPE` | `" [shape=box,style=filled,color=cyan]"` |

- main.ml2db.mulan.learn.crosseval.EvaluationResultsTable

  | Modifier and Type | Constant Field | Value |
  | --- | --- | --- |
  |  |  |  |
  | --- | --- | --- |
  | `public static final java.lang.String` | `ALGORITHM` | `"algorithm"` |
  | `public static final java.lang.String` | `END_TIMESTAMP` | `"end_time"` |
  | `public static final java.lang.String` | `EXPERIMENTS_TABLE_NAME` | `"mulan_results"` |
  | `public static final java.lang.String` | `RUN` | `"run"` |
  | `public static final java.lang.String` | `TEST_ARFF_ID` | `"test_arff_id"` |
  | `public static final java.lang.String` | `TESTSET_LABELS` | `"testset_labels"` |
  | `public static final java.lang.String` | `TRAIN_ARFF_ID` | `"train_arff_id"` |

- main.ml2db.mulan.mulanarff.MulanArffProperties

  | Modifier and Type | Constant Field | Value |
  | --- | --- | --- |
  |  |  |  |
  | --- | --- | --- |
  | `public static final java.lang.String` | `XML_QUERY_PROP` | `"xml_query"` |
- main.ml2db.mulan.mulanarff.NeighboursPrinter

  | Modifier and Type | Constant Field | Value |
  | --- | --- | --- |
  |  |  |  |
  | --- | --- | --- |
  | `public static final java.lang.String` | `ARROW_STYLE` | `" [style=bold,color=green3,penwidth=8] "` |
  | `public static final java.lang.String` | `DIRECTED_ARROW` | `" -> "` |
  | `public static final java.lang.String` | `GRAPHVIZ_HEADER` | `"graph G {\n"` |
  | `public static final int` | `NUMBER_OF_NEIGHBOURS_1` | `1` |
  | `public static final java.lang.String` | `SHAPE_GREEN_BOX` | `" [shape=box,style=filled,fillcolor=green3] "` |
  | `public static final java.lang.String` | `SHAPE_ORANGE_BOX` | `" [shape=box,style=filled,fillcolor=orange] "` |
  | `public static final java.lang.String` | `UNDIRECTED_ARROW` | `" -- "` |

- main.ml2db.mulan.predict.PredictionsExperimentTable

  | Modifier and Type | Constant Field | Value |
  | --- | --- | --- |
  |  |  |  |
  | --- | --- | --- |
  | `public static final java.lang.String` | `PREDICTION_EXPERIMENTS_TABLE_NAME` | `"prediction_experiments"` |
- main.ml2db.mulan.predict.PredictionsRecordTable

  | Modifier and Type | Constant Field | Value |
  | --- | --- | --- |
  |  |  |  |
  | --- | --- | --- |
  | `public static final java.lang.String` | `EXPERIMENT_ID` | `"experiment_id"` |
- main.ml2db.mulan.predict.PredictionsSaver

  | Modifier and Type | Constant Field | Value |
  | --- | --- | --- |
  |  |  |  |
  | --- | --- | --- |
  | `public static final java.lang.String` | `FALSE_NEGATIVES` | `"false_negatives"` |
  | `public static final java.lang.String` | `FALSE_POSITIVES` | `"false_positives"` |
  | `public static final java.lang.String` | `TRUE_POSITIVES` | `"true_positives"` |

- main.ml2db.weka.ArffProps

  | Modifier and Type | Constant Field | Value |
  | --- | --- | --- |
  |  |  |  |
  | --- | --- | --- |
  | `public static final java.lang.String` | `ARFF_DATA_PROP` | `"arff_data_file"` |
  | `public static final java.lang.String` | `ARFF_FILE_PATH_PROP` | `"arff_file_path"` |
  | `public static final java.lang.String` | `CLASS_NAME_PROP` | `"class_name"` |
  | `public static final java.lang.String` | `DATASET_NAME_PROP` | `"dataset"` |
  | `public static final java.lang.String` | `DESCRIPTION_PROP` | `"description"` |
  | `public static final java.lang.String` | `NUMBER_OF_ATTRIBUTES` | `"attributes"` |
  | `public static final java.lang.String` | `NUMBER_OF_CLASS_VALUES` | `"class_values"` |
  | `public static final int` | `NUMBER_OF_CORE_PROPERTIES` | `4` |
  | `public static final java.lang.String` | `NUMBER_OF_INSTANCES` | `"instances"` |
  | `public static final int` | `NUMBER_OF_STATISTICS_PROPERTIES` | `3` |
  | `public static final java.lang.String` | `XML_DATA_PROP` | `"xml_data_file"` |
  | `public static final java.lang.String` | `XML_FILE_PATH_PROP` | `"xml_file_path"` |
- main.ml2db.weka.ArffPropsFromFile

  | Modifier and Type | Constant Field | Value |
  | --- | --- | --- |
  |  |  |  |
  | --- | --- | --- |
  | `public static final java.lang.String` | `DATA_PROPERTIES_FILE_PATH_PROP` | `"properties_file_path"` |
  | `public static final java.lang.String` | `INSTANCE_ATTRIBUTE_QUERY_PROP` | `"instance_attribute_query"` |
  | `public static final java.lang.String` | `INSTANCE_CLASS_QUERY_PROP` | `"instance_class_query"` |
  | `public static final java.lang.String` | `TIMESTAMP_FIELD` | `"timestamp"` |
- main.ml2db.weka.ArffRecordsTable

  | Modifier and Type | Constant Field | Value |
  | --- | --- | --- |
  |  |  |  |
  | --- | --- | --- |
  | `public static final java.lang.String` | `ARFF_ID_COLUMN` | `"id"` |
  | `public static final java.lang.String` | `TABLE_NAME` | `"arff"` |
- main.ml2db.weka.AttributeFactory

  | Modifier and Type | Constant Field | Value |
  | --- | --- | --- |
  |  |  |  |
  | --- | --- | --- |
  | `public static final java.lang.String` | `BINARY_ATTRIBUTE_ABSENT_VALUE` | `"0"` |
  | `public static final java.lang.String` | `BINARY_ATTRIBUTE_PRESENT_VALUE` | `"1"` |
  | `public static final java.lang.String` | `DUMMY_VALUE` | `"dummy"` |
  | `public static final java.lang.String` | `NOMINAL` | `"nominal"` |
  | `public static final java.lang.String` | `NUMERIC` | `"numeric"` |
  | `public static final java.lang.String` | `UNKNOWN` | `"?"` |
- main.ml2db.weka.DataSetManager

  | Modifier and Type | Constant Field | Value |
  | --- | --- | --- |
  |  |  |  |
  | --- | --- | --- |
  | `public static final java.lang.String` | `DUMMY_VALUE` | `"dummy"` |
- main.ml2db.weka.InstAttValueMap

  | Modifier and Type | Constant Field | Value |
  | --- | --- | --- |
  |  |  |  |
  | --- | --- | --- |
  | `public static final java.lang.String` | `CLASS_ATTRIBUTES_TAG` | `"CA_"` |
  | `public static final java.lang.String` | `FEATURE_ATTRIBUTES_TAG` | `"FA_"` |


## test.ml2db.\*

- test.ml2db.dataharness.DataOne

  | Modifier and Type | Constant Field | Value |
  | --- | --- | --- |
  |  |  |  |
  | --- | --- | --- |
  | `public static final java.lang.String` | `arffEmptyInstance` | `"{}"` |
  | `public static final java.lang.String` | `arffInstance1` | `"{0 1,1 1,5 CA_class1}"` |
  | `public static final java.lang.String` | `arffInstance1b` | `"{0 1,1 1,5 CA_class2}"` |
  | `public static final java.lang.String` | `arffInstance2` | `"{2 1,5 CA_class3}"` |
  | `public static final java.lang.String` | `arffInstance4` | `"{3 1,5 CA_class2}"` |
  | `public static final java.lang.String` | `arffInstanceAttVoid` | `"{5 CA_class4}"` |
  | `public static final java.lang.String` | `arffInstanceClassVoid` | `"{4 1}"` |
  | `public static final java.lang.String` | `ATT1` | `"att1"` |
  | `public static final java.lang.String` | `ATT1_TO_STRING` | `"@attribute FA_att1 {0,1}"` |
  | `public static final java.lang.String` | `ATT2` | `"att2"` |
  | `public static final java.lang.String` | `ATT2_TO_STRING` | `"@attribute FA_att2 {0,1}"` |
  | `public static final java.lang.String` | `ATT3` | `"att3"` |
  | `public static final java.lang.String` | `ATT3_TO_STRING` | `"@attribute FA_att3 {0,1}"` |
  | `public static final java.lang.String` | `ATT4` | `"att4"` |
  | `public static final java.lang.String` | `ATT4_TO_STRING` | `"@attribute FA_att4 {0,1}"` |
  | `public static final java.lang.String` | `ATT5` | `"att5"` |
  | `public static final java.lang.String` | `ATT5_TO_STRING` | `"@attribute FA_att5 {0,1}"` |
  | `public static final java.lang.String` | `CA_CLASS1` | `"CA_class1"` |
  | `public static final java.lang.String` | `CA_CLASS2` | `"CA_class2"` |
  | `public static final java.lang.String` | `CA_CLASS3` | `"CA_class3"` |
  | `public static final java.lang.String` | `CA_CLASS4` | `"CA_class4"` |
  | `public static final java.lang.String` | `CLASS_TO_STRING` | `"@attribute class_name {dummy,CA_class1,CA_class2,CA_class3,CA_class4}"` |
  | `public static final java.lang.String` | `CLASS1` | `"class1"` |
  | `public static final java.lang.String` | `CLASS2` | `"class2"` |
  | `public static final java.lang.String` | `CLASS3` | `"class3"` |
  | `public static final java.lang.String` | `CLASS4` | `"class4"` |
  | `public static final java.lang.String` | `DATASET_NAME` | `"test_data_1"` |
  | `public static final java.lang.String` | `FA_ATT1` | `"FA_att1"` |
  | `public static final java.lang.String` | `FA_ATT2` | `"FA_att2"` |
  | `public static final java.lang.String` | `FA_ATT3` | `"FA_att3"` |
  | `public static final java.lang.String` | `FA_ATT4` | `"FA_att4"` |
  | `public static final java.lang.String` | `FA_ATT5` | `"FA_att5"` |
  | `public static final java.lang.String` | `INST1` | `"inst1"` |
  | `public static final java.lang.String` | `INST2` | `"inst2"` |
  | `public static final java.lang.String` | `INST3` | `"inst3"` |
  | `public static final java.lang.String` | `INST4` | `"inst4"` |
  | `public static final java.lang.String` | `INSTATTVOID` | `"inst_att_void"` |
  | `public static final java.lang.String` | `INSTCLASSATTVOID` | `"inst_class_att_void"` |
  | `public static final java.lang.String` | `INSTCLASSVOID` | `"inst_class_void"` |
- test.ml2db.dataharness.DataTwo

  | Modifier and Type | Constant Field | Value |
  | --- | --- | --- |
  |  |  |  |
  | --- | --- | --- |
  | `public static final java.lang.String` | `arffEmptyInstance` | `"{}"` |
  | `public static final java.lang.String` | `arffInstance1` | `"{0 y,1 y,5 class1}"` |
  | `public static final java.lang.String` | `arffInstance1b` | `"{0 y,1 y,5 class2}"` |
  | `public static final java.lang.String` | `arffInstance2` | `"{2 y,5 class3}"` |
  | `public static final java.lang.String` | `arffInstance4` | `"{3 y,5 class2}"` |
  | `public static final java.lang.String` | `arffInstanceAttVoid` | `"{5 class4}"` |
  | `public static final java.lang.String` | `arffInstanceClassVoid` | `"{4 y}"` |
  | `public static final java.lang.String` | `DATASET_NAME` | `"test_ec_data"` |
  | `public static final java.lang.String` | `INST1` | `"inst1"` |
  | `public static final java.lang.String` | `INST2` | `"inst2"` |
  | `public static final java.lang.String` | `INST3` | `"inst3"` |
  | `public static final java.lang.String` | `INST4` | `"inst4"` |
- test.ml2db.dataharness.TestProjectParameters

  | Modifier and Type | Constant Field | Value |
  | --- | --- | --- |
  |  |  |  |
  | --- | --- | --- |
  | `public static final java.lang.String` | `ARFF_DB_PROPS` | `"data/test/properties_files/arff_props/arff_table_db_connection.props"` |
  | `public static final java.lang.String` | `ARFF_FILES_PATH` | `"data/test/arff/"` |
  | `public static final java.lang.String` | `ARFF_PROPS_NO_XML` | `"data/test/properties_files/arff_props/test_arff_no_xml.props"` |
  | `public static final java.lang.String` | `ARFF_PROPS_NUMERIC_ATT` | `"data/test/properties_files/arff_props/test_arff_numeric_attributes.props"` |
  | `public static final java.lang.String` | `ARFF_PROPS_PATH` | `"data/test/properties_files/arff_props/"` |
  | `public static final java.lang.String` | `BASE_PATH` | `"data/test/"` |
  | `public static final boolean` | `DEBUG` | `false` |
  | `public static final java.lang.String` | `FILTER_OLD_TEST_SET` | `"data/test/properties_files/arff_props/testset_arff_for_filtering.props"` |
  | `public static final java.lang.String` | `FILTER_TRAIN_SET` | `"data/test/properties_files/arff_props/trainset_arff_for_filtering.props"` |
  | `public static final java.lang.String` | `INSTANCE_1_2` | `"data/test/properties_files/arff_props/instance_1_2.props"` |
  | `public static final java.lang.String` | `INSTANCE_1_2_EMPTY` | `"data/test/properties_files/arff_props/instance_1_2_empty.props"` |
  | `public static final java.lang.String` | `INSTANCE_3` | `"data/test/properties_files/arff_props/instance_3.props"` |
  | `public static final java.lang.String` | `ML_PROPS_PATH` | `"data/test/properties_files/machine_learning_props/"` |
  | `public static final java.lang.String` | `MODEL_FOLDER` | `"data/test/model/"` |
  | `public static final java.lang.String` | `MODELS_PATH` | `"data/test/model/"` |
  | `public static final java.lang.String` | `PLOTS_FOLDER` | `"data/test/plots/"` |
  | `public static final java.lang.String` | `PREDICTIONS_DATA` | `"data/test/predictions/data_to_test_MulanPredict/"` |
  | `public static final java.lang.String` | `PREDICTIONS_FOLDER` | `"data/test/predictions/"` |
  | `public static final java.lang.String` | `PROPS_PATH` | `"data/test/properties_files/"` |
  | `public static final java.lang.String` | `RESULTS_DIRECTORY_ACTIVE_LEARNING` | `"data/test/results/active_learning/"` |
  | `public static final java.lang.String` | `RESULTS_FOLDER` | `"data/test/results/"` |
  | `public static final java.lang.String` | `TEST_ARFF_PROPS_1` | `"data/test/properties_files/arff_props/test_arff_1.props"` |
  | `public static final java.lang.String` | `TEST_ARFF_PROPS_2` | `"data/test/properties_files/arff_props/test_arff_2.props"` |
  | `public static final java.lang.String` | `TEST_FIGURES_PATH` | `"data/test/figures"` |
  | `public static final java.lang.String` | `TEST_MACHINE_LEARNING_PROPS` | `"data/test/properties_files/machine_learning_props/mulan_test_results.props"` |

- test.ml2db.mulan.attributesfilter.AttFiltArffTest

  | Modifier and Type | Constant Field | Value |
  | --- | --- | --- |
  |  |  |  |
  | --- | --- | --- |
  | `public static final int` | `TEST_ROLE` | `1` |
  | `public static final int` | `TRAIN_ROLE` | `0` |

- test.ml2db.mulan.mulanarff.MulanArffTest

  | Modifier and Type | Constant Field | Value |
  | --- | --- | --- |
  |  |  |  |
  | --- | --- | --- |
  | `public static final java.lang.String` | `arff_EC_Instance1` | `"{0 1,1 1,2 1,3 1,8 1}"` |
  | `public static final java.lang.String` | `arffInstance1` | `"{0 1,1 1,5 1,6 1}"` |
  | `public static final java.lang.String` | `arffInstance2` | `"{2 1,7 1}"` |
  | `public static final java.lang.String` | `arffInstance4` | `"{3 1,6 1}"` |
  | `public static final java.lang.String` | `arffInstanceAttributeVoid` | `"{8 1}"` |
  | `public static final java.lang.String` | `arffInstanceClassAttributeVoid` | `"{}"` |
  | `public static final java.lang.String` | `arffInstanceClassVoid` | `"{4 1}"` |
  | `public static final java.lang.String` | `ATT_EC_1_TO_STRING` | `"@attribute CA_1.-.-.- {0,1}"` |
  | `public static final java.lang.String` | `ATT_EC_11_TO_STRING` | `"@attribute CA_1.1.-.- {0,1}"` |
  | `public static final java.lang.String` | `ATT_EC_111_TO_STRING` | `"@attribute CA_1.1.1.- {0,1}"` |
  | `public static final java.lang.String` | `ATT_EC_1111_TO_STRING` | `"@attribute CA_1.1.1.1 {0,1}"` |
  | `public static final java.lang.String` | `ATT_EC_2_TO_STRING` | `"@attribute CA_2.-.-.- {0,1}"` |
  | `public static final java.lang.String` | `ATT_EC_22_TO_STRING` | `"@attribute CA_2.2.-.- {0,1}"` |
  | `public static final java.lang.String` | `ATT_EC_222_TO_STRING` | `"@attribute CA_2.2.2.- {0,1}"` |
  | `public static final java.lang.String` | `ATT_EC_2222_TO_STRING` | `"@attribute CA_2.2.2.2 {0,1}"` |
  | `public static final java.lang.String` | `ATT1_TO_STRING` | `"@attribute FA_att1 {0,1}"` |
  | `public static final java.lang.String` | `ATT2_TO_STRING` | `"@attribute FA_att2 {0,1}"` |
  | `public static final java.lang.String` | `ATT3_TO_STRING` | `"@attribute FA_att3 {0,1}"` |
  | `public static final java.lang.String` | `ATT4_TO_STRING` | `"@attribute FA_att4 {0,1}"` |
  | `public static final java.lang.String` | `ATT5_TO_STRING` | `"@attribute FA_att5 {0,1}"` |

- Overview
- Package
- Class
- Use
- Tree
- Deprecated
- Index
- Help

- Prev
- Next

- Frames
- No Frames

- All Classes
